# One Year Trajectory of Caregiver Burden in Parkinson’s Disease and Analysis of Gender-Specific Aspects

**DOI:** 10.3390/brainsci11030295

**Published:** 2021-02-26

**Authors:** Martin Klietz, Hannah von Eichel, Theresa Schnur, Selma Staege, Günter U. Höglinger, Florian Wegner, Stephanie Stiel

**Affiliations:** 1 Department of Neurology, Hannover Medical School, 30625 Hannover, Germany; Hannah.C.V.Eichel@stud.mh-hannover.de (H.v.E.); Schnur.Theresa@stud.mh-hannover.de (T.S.); Staege.Selma@mh-hannover.de (S.S.); Hoeglinger.Guenter@mh-hannover.de (G.U.H.); Wegner.Florian@mh-hannover.de (F.W.); 2 Center for Systems Neuroscience, 30625 Hannover, Germany; 3 Institute for General Practice, Hannover Medical School, 30625 Hannover, Germany; Stiel.Stephanie@mh-hannover.de

**Keywords:** Parkinson’s disease, caregiver burden, Parkinson’s disease caregiver burden questionnaire, gender, health-related quality of life, depression

## Abstract

Parkinson’s disease (PD) is a slowly progressive neurodegenerative movement disorder that leads to impairments in activities of daily living. In addition to reducing patients’ quality of life, this disease also affects caregivers’ well-being. Until recently, caregiver burden was mainly assessed by generic questionnaires, which do not take the characteristics of the chronic disease into consideration. In the case of PD, this issue has been addressed by the introduction of the “Parkinson’s disease caregiver burden” questionnaire (PDCB). Data on longitudinal trajectories of caregiver burden are still missing in the literature. In this study, we assessed the one-year trajectory of caregiver burden by the PDCB as a disease-specific questionnaire. Further, gender-specific aspects of caregiver burden were analyzed by applying a caregiver task questionnaire. PDCB total score (*n* = 84 patients and caregivers) did not significantly change from baseline (30.4) to one year at follow-up (31.5). No significant difference was detected between female and male caregivers in global burden and-specific caregiver tasks. Our data showed only a mild increase of caregiver burden in the timeframe of one year. Gender-specific differences do not seem to impact-specific caregiver tasks in the presented study population.

## 1. Introduction

Parkinson’s disease (PD) is a progressive neurodegenerative disease leading to an enormous reduction of quality of life and increasing the need for care [1,2]. A huge amount of care for advanced PD patients is provided by informal caregivers [3]. These caregivers experience a large burden, which can lead to physical and psychological complaints [4]. Furthermore, an excessive burden can cause depression and, eventually, lead to burnout of the caregiver, which may result in institutionalization of the PD patient [5].

Measurement of caregiver burden is complex and time-consuming. Generally, caregiver burden in PD is assessed by generic questionnaires. These tests do not explicitly evaluate all specific aspects of caregiver burden in PD, although the course of PD is different from other geriatric diseases, e.g., due to various motor and non-motor symptoms and long disease duration with progressive impairments in the activities of daily living [6,7]. The recently developed and validated Parkinson’s disease caregiver burden questionnaire (PDCB) offers a special opportunity to study disease-specific aspects of caregiver burden in PD [8]. However, no longitudinal data were obtained by using the PDCB for the assessment of PD caregiver burden. Further, the impact of caregivers’ gender on-specific aspects of caregiver burden is still not known in PD.

This study aims to characterize the one-year-longitudinal trajectory of caregiver burden in a German cohort of PD caregivers’ overall disease stages and analyses gender-specific aspects of burden due to caregiving.

## 2. Materials and Methods

### 2.1. Participants

The local ethics committee of Hannover Medical School provided ethical approval (no. 3178-2016, Amendment in 2018). At baseline (03/2019), our sample included 124 PD patients and their 124 primary caregivers, who have all given their written informed consent. After one year (03/2020), we contacted these patients and caregivers again for a follow-up investigation. Questionnaires were sent via mail, and, in case of no answer, participants were contacted to get further information about their drop out. Of the 124 initial participants, 89 responded in the follow-up investigation. Participants who did not take part in the follow-up were also excluded from the baseline. The PD patients included in the study had been diagnosed with a neurologically determined PD for at least one year at baseline. Only patients who met the Movement Disorder Society (MDS) clinical diagnostic criteria for PD [9], lived at home and had not been diagnosed with atypical Parkinsonism were included in the analysis together with their primary caregiver (mostly spouses). In the case of multiple caregivers, only one caregiver spending the most time with the patient and handling most of the care issues was considered in the study. Professional caregivers were excluded. Dropouts of the study were either not contactable, had been transferred into institutional care or the caregiver was not able to complete the follow-up questionnaire. Participants did not receive any financial compensation for participating in the study.

### 2.2. Measures

The patients’ PD impairments were classified with the Hoehn and Yahr stage, which has a minimum of 1 point (unilateral symptoms) and a maximum of 5 points (confinement to bed or wheelchair) [10].

To assess health-related quality of life (HR-QoL), patients filled out the Parkinson’s disease quality of life questionnaire 8 (PDQ-8) [11], which evaluates patients’ functioning and well-being in 8 different domains. Patients were examined using 8 questions on a 5-point Linkert scale ranging from 0 to 4 with higher values denoting lower HR-QoL. The total score, which ranged from 0 to 32 points, was then converted in the percentage of HR-QoL restrictions. A high percentage point indicated more severe HR-QoL restrictions of the PD patient. To avoid anosognosia and ensure correct results, caregivers were asked to support patients who had a disease-related impairment in completing the PDQ-8 (as also done in [12,13,14]).

We asked the PD patients to fill out the Movement Disorders Society unified Parkinson’s disease rating scale (MDS-UPDRS) part I and part II [15,16] to evaluate the patients’ impairment of daily living regarding non-motor symptoms (part I) and motor impairments (part II).

Patient and caregiver depressive symptoms were assessed by Beck’s depression inventory [17] (BDI, depressive symptoms ranging from 0–63 points: 0–13 no depression, 14–19 mild depression, 20–28 moderate depression, 29–63 severe depression).

General information, such as age, patients´ disease duration and daily amount of care time dedicated to the patients, were also collected from the caregivers.

A validated German version of the Parkinson´s disease caregiver burden questionnaire (PDCB) was then distributed to the caregivers [8,14]. The questionnaire includes 20 items on a 5-point Likert scale (0 to 4), and the participants can score a maximum of 80 points on this first part of the questionnaire. In the second part, participants were also asked to rate their global burden as a caregiver on a scale from 0 to 100. Finally, points from the first part and 20% of the global burden were summed up for the total caregiver burden score; the highest possible burden corresponds to the maximum score of 100. These 20 questions can also be grouped into 7 subscales [8]. In each subscale, the maximum points available depending on the number of questions included in the subscale. By summing up the points of the individual questions, one obtains the number of points in each subscale.

The short-form 36 health survey (SF-36) [18] measures the HR-QoL of the caregivers across eight dimensions. The subscale scores are given as a percentage, with a score of 0 indicating maximum impairment and a score of 100 indicating the absence of any reported impairment. To account for the high correlation between the subscale scores, we calculated an average score across the eight scales [19].

Caregivers answered a caregiver task questionnaire, which includes 12 items describing different caregiver tasks, adapted from Rowland et al. [20]. Each item has a score ranging from 0 to 10 points, with 0 representing no burden and 10 an unbearable burden. This questionnaire aims to determine the tasks which are considered to be the most stressful for the caregivers. The total score results from the sum of all individual items and has a minimum of 0 and a maximum of 120 points.

### 2.3. Analyses

Data were displayed in mean and standard deviation (SD) for descriptive analysis. Values with *p* < 0.05 were considered statistically significant. A Wilcoxon test was used to determine the significance of changes between t_0_ (baseline) and t_1_ (one-year follow-up) for the PDCB and its subscales. Gender-specific differences (male/female) for the patient and caregiver characteristics were calculated by using t-tests for interval scales (such as sum and total scores from PDQ-8, MDS-UPDRS part I and part II, BDI, Caregiving hours per day, PDCB, BDI, and SF-36 total) and a chi-squared test for the ordinal scale Hoehn and Yahr stage). Analyses were carried out using SPSS v25.0 (IBM, Armonk, NY, USA).

## 3. Results

### 3.1. Patient and Caregiver Characteristics

The demographic and clinical characteristics of 89 patients with PD and their 84 caregivers from baseline and one-year follow-up are shown in Table 1. Over one year, the outcome variables have slightly changed. Regarding the caregiver burden as the main interest of our study, caregivers did not develop significantly more caregiver burden measured by the PDCB over one year (Table 2). The patients’ HR-QoL (measured by PDQ-8:33.2 ± 21.2 at t_0_ and 34.8 ± 17.8 at t_1_), motor impairment of daily living (UPDRS part II: 17.0 ± 11.0 at t_0_ and 17.7 ± 10.6 at t_1_) and depression (BDI: 11.4 ± 7.6 at t_0_ and 12.0 ± 8.0 at t_1_) remained stable in the time-span of one year. Stable results were also observed for the caregivers who spent 5.3 ± 6.6 h per day caregiving the patients at t_0_ and 5.5 ± 6.5 h at t_1_ and reported an average depression score of 9.4 ± 6.8 at t_0_ and 9.2 ± 6.8 at t_1_.

### 3.2. Longitudinal Trajectory of Caregiver Burden in Parkinson’s Disease

A Wilcoxon regression was performed to evaluate whether the PDCB total score or one of the subscales differed at one-year follow-up. The PDCB total score has not changed significantly (30.4 ± 16.4 at t_0_ and 31.5 ± 16.0 at t_1_), neither have its subscales nor the PDCB global burden scale (31.6 ± 26.3 at t_0_ to 33.3 ± 25.6 at t_1_). By normalizing the score of the PDCB subscales with the number of questions in each subscale, we found the lowest value in the subscale “physical burden” (1.6 ± 1.6 at t_0_ and 1.6 ± 1.6 at t_1_), whereas the highest burden was detected in the subscale “patient and self-relationship” (4.5 ± 2.9 at t_0_ and 4.9 ± 3.2 at t_1_). All subscale scores remained constant over the one-year follow-up.

In Appendix A and Appendix B, recruitment issues were carefully addressed to clarify differences between participants at the follow-up and dropouts, including reasons for dropping out. The population which dropped out at the follow-up was more burdened, displayed more depressive symptoms, spent more hours day caregiving and experienced worse HR-QoL.

### 3.3. Impact of Caregiver Gender on Caregiver Burden in Parkinson’s Disease

Patient and caregiver follow-up-characteristics were separately analyzed according to gender (Table 3). To examine gender-specific differences in caregiver burden, we carried out a *t*-test. No significant difference between female and male patients could be found, except for higher PD severity in the female group (Hoehn and Yahr chi = 0.012) and worse HR-QoL of the female PD patients (PDQ-8 I-test 0.030). Only age was significantly higher in male than in female caregivers (*p* = 0.005), while no difference could be detected in the caregiver burden (PDCB), the daily caregiving-hours, caregivers’ depression (BDI) or their HR-QoL (SF-36).

A caregiver task-questionnaire was adapted in order to investigate which tasks burden the caregiver the most and which ones show a gender-specific difference (Table 4). Caregivers felt most burdened by providing social and emotional support and by taking part in decision-making. The least burdening tasks were mobilizing or positioning the patient and alleviating symptoms. Overall, no difference was detected between female and male caregiver burden.

## 4. Discussion

In the present study, the longitudinal trajectory of caregiver burden was assessed in a German cohort of PD patients and their caregivers. Over the time-span of one-year, caregiver burden measured by the disease-specific questionnaire PDCB remained constant. Furthermore, the gender-specific burden was analyzed. In the study population, no differences in overall PDCB values between genders were detected. “Providing social and emotional support” and “taking part in decision-making” were the most burdensome activities as stated by the caregivers. Concerning gender-specific differences, no activity reached a statistical significance.

### 4.1. Longitudinal Trajectory of the Parkinson’s Disease Caregiver Burden

Caregiver burden is a complex construct of patient and caregiver factors resulting in depressive symptoms and reduced HR-QoL of the caregiver. There is a large body of evidence on how specific symptoms contribute to caregiver burden [21]. However, longitudinal data of observational studies were missing in the literature. In our one-year-trajectory study, no significant change could be shown by using the disease-specific PDCB questionnaire for the assessment of caregiver burden [8].

In several treatment trials for levodopa/carbidopa intestinal gel [22] or deep brain stimulation [23] of PD patients, caregiver burden was used as a secondary end-point. Lopiano et al. did not find a significant change of caregiver burden over a period of 3 years in PD patients treated with levodopa/carbidopa intestinal gel by using a generic caregiver burden questionnaire [24]. Another group reported data of 12 PD patients and their caregivers before and 6 months after initiation of levodopa/carbidopa intestinal gel therapy [22], showing a significant reduction of caregiver burden and other related caregiver outcome parameters measured by a generic caregiver burden questionnaire. Mosley et al. reported data from PD patients before and after subthalamic deep brain stimulation [23] without a significant difference between caregiver burden at baseline and last follow-up after 26 weeks using the Zarrit burden inventory [23].

However, to our knowledge, no data on the longitudinal trajectory of caregiver burden in PD is available from observational studies without changes in PD therapy. In most of the longitudinal studies, a generic caregiver burden questionnaire was used to assess burdened caregivers [25]. However, these questionnaires were not disease-specific and might neglect PD-specific features of caregiver burden, contrasting a disease-specific questionnaire [26,27,28].

Usage of the PDCB offers new opportunities to assess caregiver burden related to specific PD symptoms [13]. Interestingly, none of the subitems of the PDCB (e.g., physical burden, sleep disturbance, patient symptoms, responsibilities, patient medications, social burden and patient and self-relationship) showed a significant increase over one year in our present study. One reason for this might be that PD is a slowly progressive disease with only slightly increased mortality compared to non-PD elderly [29].

Further, these data must be interpreted with caution, as a third of the initial participants did not participate in the follow-up assessment. Greenwell et al. also identified the problem of dropouts at follow-up in PD caregivers [26]. Generally, burdened caregivers do not want to participate in observational studies or discontinue participation in the study because of severe burdens. These patients and their caregivers were therefore often neglected by studies. A recent international study focused on late-stage PD patients and their caregivers, describing moderate caregiver burden. Interestingly, this burden was not significantly different between patients in institutional care and outpatient PD patients [30]. Overall, caregiver burden measured by the Zarrit burden inventory showed stable results in several European countries in this study [25,30]. Other studies of different neurodegenerative diseases have reported a significant increase in caregiver burden over time. Connors et al. showed a significant increase in burden in relatives of people with dementia over a period of three years [31]. An increase of caregiver burden over a time period of 9 months was also shown for caregivers of amyotrophic lateral sclerosis (ALS) patients [32].

### 4.2. Impact of Gender on Caregiver Burden in Parkinson’s Disease

In the present study, the asymmetrical gender distribution of PD patients was observed. The larger fraction of PD patients were males, and, as most caregivers were their spouses, the caregiver group included more females, which is consistent with other studies of our group [12,13,14]. This is in line with the generally asymmetrical gender distribution in PD with a 1.6:1 relation of male to female patients [33]. Despite this fact, women with PD often experienced a more malignant disease course with faster progression and higher mortality rates [34]. In addition, female patients display several non-motor symptoms in a more severe manner, like fatigue, restless legs, constipation, depression, pain, impairment of taste or smell, weight changes and excessive sweating [35]. Further, Balzer-Geldsetzer et al. suggested female sex as a predictor for worse HR-QoL in the subdomains physical function and socio-emotional QoL, whereas male patients were more affected by cognitive restrictions in HR-QoL [36].

Based on these preconditions and since clear differences between the sexes in the course of PD have been described [37], the present study aimed to detect differences in gender-specific caregiver burden using a PD-specific caregiver burden questionnaire. However, no significant differences between female and male caregivers were detected in the study sample. In a further analysis, caregiver-specific tasks were evaluated in a gender-specific manner [20]. In none of the specific caregiver tasks (e.g., shopping, housekeeping, preparing/serving meal, general body care of the patient, mobilizing/positioning the patient, helping the patient during the night, alleviating symptoms, providing social/emotional support, gathering information, taking part in decision-making, managing administrative tasks, accompanying the patient to appointments) gender-specific burden could be detected. Regarding caregiver tasks, McLaughlin et al. showed that caregivers must adapt to many tasks of daily living of the patient during disease progression, which leads to progressive caregiver burden [28].

In a recent study, Lee et al. could also not identify gender-specific differences in the burden of PD caregivers [38]. Furthermore, this was confirmed by a community-based study from India with 150 PD patients and their informal caregivers [39]. In a more detailed study by Balash et al., female caregivers of PD patients reported twice as often as males to feel exhaustion and impaired health status from caregiving [40]. Moreover, social constraints and time limitations were more present in female caregivers of PD patients [40]. Another study by Grün et al. found that female caregivers caring for male patients subjectively experienced higher caregiver burden [41]. Additionally, female caregivers of patients with PD had a reduced quality of life compared to male caregivers [42]. However, Hooker et al. reported no gender differences in a cohort of 87 PD patients and their caregivers, contrary to more caregiver burden of female caregivers in a cohort of Alzheimer’s disease [43].

Interestingly, dystonia affects more female patients, and therefore, a larger proportion of male caregivers were evaluated in a recent study concerning caregiver burden, which was not pronounced under botulinum toxin therapy of the patients [19]. In a meta-analysis of predictors for general caregiver burden in chronic illnesses, Adelman et al. identified female sex as an individual risk factor [44]. In another study about people with multiple chronic conditions and their caregivers, the male sex of the caregiver was associated with the less impaired mental health of the caregiver [44,45]. The female sex of the caregiver could also be identified as an individual risk-factor for caregiver burden in a longitudinal cohort of caregivers of people with dementia [31].

With regard to gender-specific differences in other neurodegenerative disorders, such as ALS, Tramonti et al. reported a significantly higher burden among female caregivers of ALS patients [46]. In contrast, in another study on caregiver burden in ALS, caregivers did not show a difference between the sexes [47].

Combining the presented evidence, the gender of the caregiver may have some impact on caregiver burden. However, in our study, no differences in general and task-specific burden could be detected for caregivers of PD patients. Reasons for this stable outcome may be a successful medical therapy of the PD patients, the moderate number of participants, the drop out of most burdened caregivers or the asymmetrical gender distribution of caregivers.

### 4.3. Limitations

This study presents longitudinal data on caregiver burden in a German PD cohort. However, at the follow-up after one year, only 67.7% of the PD patients and caregivers participated in the second assessment. Unfortunately, a high proportion of severely affected patients and burdened caregivers refused a second assessment due to the huge amount of burden. This led to a selection bias in favor of the caregivers with less burden because the others no longer took part in the study. Although the variance of our data is quite large, most fining did not find significance. Larger sample size incomparable future studies may contribute to more significant findings. Further, since PD affects more males than females, we observed more male PD patients and female caregivers. Male caregivers were significantly older in our study cohort, which may also bias our results. Moreover, we were not able to match male and female caregivers in a one-to-one ratio for better comparability (data not shown). This matching should be considered in future studies with more participants. Hence, longitudinal prospective interventional studies are needed to determine the effect of specific potentially multidimensional interventions on the caregiver burden of female and male caregivers in the future. This study also did not evaluate patients’ comorbidities and medications [48,49].

## 5. Conclusions

Caregiver burden is a highly relevant issue in the care of PD patients. To our knowledge, this is the first study providing longitudinal data on caregiver burden measured by the PDCB. The longitudinal progression of caregiver burden in one year seems to be slow, as the disease also progresses slowly. Gender of the caregiver may contribute to the experienced caregiver burden; however, no significant differences in general and task-specific burden could be detected in the presented cohort. Despite negative results in this study, gender aspects of PD patients and their caregivers seem to be clinically relevant and should be addressed in future studies.

## Figures and Tables

**Table 1:** Patient (*n* = 89, *n* = 34 females) and caregiver (*n* = 84, *n* = 54 females) characteristics.

	Mean ± SD (t_0_)	Mean ± SD (t_1_)
**PD patients**		
Age (years)	68.3 ± 10.1	68.7 ± 10.1
Disease duration (years)	10.8 ± 6.2	11.7 ± 6.5
Hoehn and Yahr stage	2.8 ± 1.0	2.9 ± 1.0
PDQ-8	33.2 ± 21.2	34.8 ± 17.8
MDS-UPDRS part I	11.0 ± 6.7	10.0 ± 5.1
MDS-UPDRS part II	17.00 ± 11.0	17.7 ± 10.6
BDI	11.4 ± 7.6	12.0 ± 8.0
**Caregivers**		
Age (years)	65.1 ± 10.3	66.2 ± 10.6
Caregiving hours per day	5.3 ± 6.5	5.5 ± 6.5
PDCB	30.4 ± 16.4	31.5 ± 16.0
BDI	9.4 ± 6.8	9.2 ± 6.8
SF-36 total	67.7 ± 18.6	64.0 ± 19.6

Abbreviations: BDI, Beck’s depression inventory; MDS-UPDRS, Movement Disorders Society Unified Parkinson’s Disease Rating Scale; PD, Parkinson’s disease; PDCB, Parkinson’s disease caregiver burden questionnaire; PDQ-8, Parkinson’s disease questionnaire 8; SD, standard deviation; SF-36, short-form 36 health survey; t_0_, baseline (03/2019); t_1_, follow-up (03/2020).

**Table 2:** Longitudinal course of the “Parkinson’s disease caregiver burden” questionnaire (PDCB) subscales.

PDCB Subscale	Mean ± SD (t_0_)	Mean ± SD (t_1_)	Wilcoxon Test
Physical burden (2)	1.6 ± 1.6	1.6 ± 1.6	0.71
Sleep disruption (2)	2.5 ± 2.3	2.7 ± 2.1	0.69
Patient symptoms (5)	6.9 ± 4.2	7.1 ± 4.1	0.87
Responsibilities (3)	3.8 ± 2.9	3.9 ± 2.5	0.68
Patient medications (2)	2.4 ± 2.1	2.4 ± 2.0	0.815
Social burden (3)	2.5 ± 2.3	3.0 ± 2.3	0.17
Patient + self-relationship (3)	4.5 ± 2.9	4.9 ± 3.2	0.35
PDCB global burden scale	31.6 ± 26.3	33.3 ± 25.6	0.31
PDCB total	30.4 ± 16.4	31.5 ± 16.0	0.44

The number of questions included in each subscale is shown in brackets. Abbreviations: PDCB, Parkinson’s disease caregiver burden inventory; SD, standard deviation; t_0_, baseline (03/2019); t_1_, follow-up (03/2020).

**Table 3:** Patient and caregiver characteristics, separated by gender.

	Mean ± SD Female (*n* = 33)	Mean ± SD Male (*n* = 54)	*p* (*t*-Test)	Chi-Squared
**PD patients**				
Age (years)	69.5 ± 11.1	68.2 ± 9.5	0.565	
Disease duration (years)	12.6 ± 7.9	9.57 ± 5.4	0.079	
Hoehn and Yahr stage	3.4 ± 0.8	2. ± 1.0		0.012 *
PDQ-8	40.0 ± 15.8	31.5 ± 18.4	0.030 *	
MDS-UPDRS part I	10.5 ± 4.7	9.6 ± 5.3	0.421	
MDS-UPDRS part II	19.9 ± 11.5	16.4 ± 9.8	0.139	
BDI	14.0 ± 7.8	10.7 ± 8.0	0.062	
**Caregivers**	**Mean ± SD** **Female (*n* = 54)**	**Mean ± SD** **Male (*n* = 30)**	***p*** **(*t*-Test)**	**Chi-Squared**
Age (years)	63.9 ± 10.0	70.6 ± 10.5	0.005 **	
Caregiving hours per day	4.8 ± 6.1	7.0 ± 7.0	0.158	
PDCB	31.4 ± 15.9	31.3 ± 16.3	0.984	
BDI	9.8 ± 7.0	7.6 ± 6.0	0.161	
SF—36 total	64.4 ± 18.6	63.6 ± 21.8	0.847	

Abbreviations: BDI, Beck’s depression inventory; MDS-UPDRS, Movement Disorders Society unified Parkinson’s disease rating scale; PD, Parkinson’s disease; PDCB, Parkinson’s disease caregiver burden questionnaire; PDQ-8, Parkinson’s disease questionnaire 8; SD, standard deviation; SF-36, short-form 36 health survey. * indicates significance at *p* < 0.05; ** indicate significance at *p* < 0.01.

**Table 4:** Caregiver task questionnaire.

Caregiver Task Questionnaire	Mean ± SD	Mean ± SD Female (*n* = 52)	Mean ± SD Male (*n* = 30)	chi-Squared (Female vs. Male)
Total score	31.0 ± 25.8	29.9 ± 25.2	33.5 ± 27.5	0.821
Shopping	2.2 ± 2.3	1.92 ± 2.3	2.5 ± 2.2	0.643
Housekeeping (e.g., cleaning, ironing)	2.8 ± 2.8	2.6 ± 2.8	3.3 ± 2.7	0.302
Preparing/serving of meals	2.4 ± 2.7	2.1 ± 2.6	2.8 ± 2.8	0.229
General body care (e.g., dressing/undressing, showering, toilet)	2.4 ± 3.0	2.1 ± 2.8	3.0 ± 3.2	0.28
Mobilizing or positioning the patient	1.8 ± 2.8	1.5 ± 2.7	2.3 ± 3.0	0.441
Helping patient during the night	2.0 ± 3.0	1.6 ± 2.8	2.9 ± 3.3	0.142
Alleviating symptoms (e.g., on-demand medication)	2.0 ± 2.5	1.9 ± 2.6	2.1 ± 2.2	0.629
Providing social/emotional support	3.9 ± 2.8	4.0 ± 3.0	3.6 ± 2.5	0.428
Gathering information	2.4 ± 2.5	2.7 ± 2.7	1.8 ± 2.1	0.163
Taking part in decision-making	3.5 ± 3.0	3.8 ± 3.0	3.2 ± 2.9	0.454
Managing administrative tasks (e.g., health insurance)	2.9 ± 3.1	3.0 ± 3.2	2.8 ± 2.3	0.36
Taking/accompanying the patient to, e.g., a doctor	2.5 ± 3.0	2.3 ± 2.9	2.9 ± 3.1	0.29

Each subitem has a point value from a minimum of 0 and a maximum of 10 points, with 0 representing no burden and 10 representing an unbearable burden. The total score is obtained by adding the points of all answers. Abbreviations: SD, standard deviation.

## Data Availability

Data are available on reasonable request to MK as the corresponding author.

## Appendix A

**Table A1:** Comparison of baseline data of the dropouts with baseline date of the caregiver with completed follow-up.

	Mean ± SD (*n* = 84 with Follow-Up)	Mean ± SD (*n* = 32 Drop-Outs)
**Caregivers (at t_0_)**		
Age (years)	65.1 ± 10.3	66.01 ± 13.0
Caregiving hours per day	5.3 ± 6.5	6.9 ± 6.2
PDCB	30.4 ± 16.4	37.8 ± 17.4
BDI	9.4 ± 6.8	11.0 ± 8.5
SF—36 total	67.7 ± 18.6	45.8 ± 33.8

Abbreviation: BDI, Beck’s depression inventory; PDCB, Parkinson’s disease caregiver burden questionnaire; SD, standard deviation; SF-36, short-form 36 health survey.

## Appendix B

**Table A2:** Reasons for drop out.

Reason for Dropping Out	*n*
Unreachable via mail/phone	21
Very stressed at home (questioned by phone)	8
Patient was no longer interested (vs. caregiver)	3
Caregiver was no longer interested (vs. patient)	3
Death of patient	2
Nursing home	2
Changed diagnosis (atypical Parkinsonism)	1

Drop-outs: *n* = 40 (t_1_ *n* = 84; t_0_ *n* = 124).
